# Does a No-Take Marine Protected Area Benefit Seahorses?

**DOI:** 10.1371/journal.pone.0105462

**Published:** 2014-08-19

**Authors:** David Harasti, Keith Martin-Smith, William Gladstone

**Affiliations:** 1 Fisheries Research, Marine Ecosystems, NSW Department of Primary Industries, Nelson Bay, New South Wales, Australia; 2 School of Zoology, University of Tasmania, Hobart, Tasmania; 3 School of the Environment, University of Technology, Sydney, New South Wales, Australia; University of Western Sydney, Australia

## Abstract

Seahorses are iconic charismatic species that are often used to ‘champion’ marine conservation causes around the world. As they are threatened in many countries by over-exploitation and habitat loss, marine protected areas (MPAs) could help with their protection and recovery. MPAs may conserve seahorses through protecting essential habitats and removing fishing pressures. Populations of White's seahorse, *Hippocampus whitei*, a species endemic to New South Wales, Australia, were monitored monthly from 2006 to 2009 using diver surveys at two sites within a no-take marine protected areas established in 1983, and at two control sites outside the no-take MPA sites. Predators of *H. whitei* were also identified and monitored. *Hippocampus whitei* were more abundant at the control sites. Seahorse predators (3 species of fish and 2 species of octopus) were more abundant within the no-take MPA sites. Seahorse and predator abundances were negatively correlated. Substantial variability in the seahorse population at one of the control sites reinforced the importance of long-term monitoring and use of multiple control sites to assess the outcomes of MPAs for seahorses. MPAs should be used cautiously to conserve seahorse populations as there is the risk of a negative impact through increased predator abundance.

## Introduction

Human uses of the marine environment have caused declines in species worldwide [1]. Over-fishing, pollution, introduction of invasive species, climate change and habitat loss continue to threaten marine species [2]. It has been estimated that the global abundance of marine fishes has declined ∼38% between 1970 and 2007 [3] and the IUCN Red List has approximately 800 marine fish species listed as threatened”. One group of fishes, the seahorses (*Hippocampus* spp.) of the family Syngnathidae, have 11 species assessed as threatened on the IUCN Red List. In several countries they have been over-harvested for traditional medicines, curios and the aquarium trade and several species face population declines as a result of loss of essential habitats and over-fishing [5], [6]. Concerns over the unsustainable trade in seahorses led to them being listed on Appendix II of the Convention on International Trade in Endangered Species (CITES) [6]. Appendix II still allows trade in *Hippocampus* spp.; however, exporting countries must be able to certify that export of seahorses is not causing a decline or damage to wild populations.

Various management options have been proposed or implemented to protect *Hippocampus* spp. in the wild including the application of minimum size limits [7], implementation of temporary fishing closures during recruitment periods [8], the protection of essential habitats [6], providing seahorses with a conservation status prohibiting collection [9], and the implementation of no-take marine protected areas (MPAs) [8], [10]–[12].

The benefits of MPAs for conserving marine biodiversity are well documented [13]–[16]; however, the potential benefit of MPAs for conserving seahorse populations is relatively unknown. It has been suggested that *Hippocampus* spp. with small over-lapping home ranges would benefit from the creation of small scale no-take MPAs [17] by protecting critical spawning biomasses [18]. The creation of no-take MPAs would also contribute towards conserving seahorse habitats by removing damaging processes, such as destructive fishing practises including dynamite fishing [11] and demersal seine netting [19].

As seahorses are charismatic species that garner considerable public support, it has been suggested they could be used as flagship species to assist with the protection of marine biodiversity around the world [6]. It has been shown that selecting MPAs for estuarine seagrass habitats, based on the density and assemblage variations of syngnathids, would benefit other fish species [20]. Seahorses have been used as a flagship marine species to help establish MPAs in the Philippines; however, the MPAs had no significant effect on seahorse densities and little effect on seahorse size [21]. In this example, the removal of fishing from the MPA did not increase densities of seahorses. This may have been because of poor habitat quality within the MPA, the biology of seahorses, and the small population sizes of seahorses outside the MPA to supply the MPA [21]. Calls for MPAs to be used generally for syngnathid conservation should be treated cautiously. The biological attributes of syngnathids, such as limited movement and strong site fidelity [22], small home range [17], early reproduction [23], and (for some species) lack of a dispersive pelagic larval phase [24], suggest that local populations are likely to respond positively to an MPA. However, there are other reasons why MPAs may not be effective for syngnathids, including specific habitat preferences of all life stages of syngnathids not being met within an MPA [21], habitat changes that follow MPA establishment leading to a decline in the availability of preferred habitat [25]–[26], larval dispersal by some species limiting opportunities for local recruitment and population replenishment [27], and the build-up of predators within an MPA causing a decline in prey species [25], potentially including syngnathids. In addition, the effectiveness of an MPA for syngnathids might be compromised by activities occurring outside the boundaries that affect habitats within the MPA, such as pollution [21]. To date, apart from Yasué *et al*. (2012), there have been no studies that have specifically tested the effects of an MPA for syngnathids.

The aim of this study was to assess the benefits of no-take MPAs on seahorses. This was done by quantifying the relative abundance of the White's seahorse *Hippocampus whitei* within multiple no-take MPAs and multiple control sites, by identifying and quantifying predators of *H. whitei*, and testing for correlations between the abundance of predators and *H. whitei*. *Hippocampus whitei* is a medium-sized seahorse (maximum length (*L*_T_) of 162 mm) that is considered endemic to several estuaries along the New South Wales (NSW) coast [23] and is protected under NSW fisheries legislation ensuring it cannot be taken from the wild [9]. The species exhibits initial rapid growth, reaches sexually maturity at approximately 6 mo and has a lifespan in the wild of 5–6 yr [23]. It occurs in a range of habitats including artificial structures [29], sponge gardens [24] and seagrass habitats [17].

## Materials and Methods

### Study Sites

**Figure 1:**
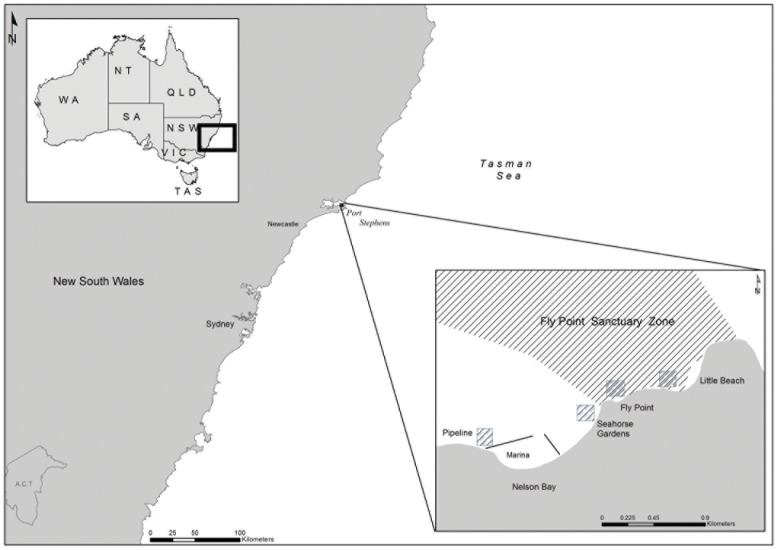
Location of study sites, Port Stephens, New South Wales – Australia.

This study was undertaken at four sites near Nelson Bay in the Port Stephens-Great Lakes Marine Park in Port Stephens, NSW, Australia (32°43′04.63′′S, 152°08′29.27′′E) (Figure 1). Each site was approximately 6000 m^2^ and ranged in depth from 2–13 m with a variety of habitat types, such as *Dendronephthya australis* soft coral, *Posidonia australis* seagrass and sponge gardens, located at each of the sites. Two of the sites (Fly Point and Little Beach) are located within the Fly Point Sanctuary Zone, a no-take zone that has been protected since 1983 with all forms of fishing excluded. The other two sites (Pipeline and Seahorse Gardens) are located in a Habitat Protection Zone, which has restrictions on commercial fishing activities such as no trawling whilst fishing and anchoring are permitted, and both are popular fishing locations (personal observations). Habitats across the four sites consisted of sponge, soft coral and seagrass habitats and it was found that there was no significant difference in habitat availability amongst three of the sites (Pipeline, Seahorse Gardens and Little Beach) [30]. Fly Point was found to contain significantly more available habitat for seahorses, as this site had the most extensive sponge garden habitat and the least amount of sand (Harasti *unpublished data*). The research undertaken in this project was done in accordance with NSW DPI Animal Care and Ethics Committee (ACEC) permit 01/05 and University of Newcastle ACEC permit 9610708.

### Relative abundance of *H. whitei*

The hypothesis that seahorse abundance would differ between the sanctuary and non-sanctuary sites was tested with data gathered during monthly surveys of each site between January 2006 and December 2009 (n = 48 monthly surveys). Seahorse abundance in each site was assessed with a 60 min random roving diver search [31], which involved the observer (DH) haphazardly swimming over the site searching for seahorses amongst the various habitats while swimming at a constant speed. To minimise problems associated with non-independence, the start and end point varied from survey to survey. When a seahorse was encountered, it was classified as male, female or juvenile. Adult males were determined by the presence of a brood pouch whilst females lacked a brood pouch and were greater than L*_T_* 75 mm. Juveniles were considered less than L*_T_* 75 mm as ∼75 mm was found to be the mean size for sexual maturity for *H. whitei* in Port Stephens [23].

### Pilot study

To determine if time of day affected the observability of *H. whitei* at the sites, a pilot study was done to test the null hypothesis that *H. whitei* abundance would not differ between day and night, as has been found for another similar sized seahorse *H. comes* that was considered to be easier to detect at night [32]. The pilot study involved conducting a 60 min diver search (as described above) at the site during daylight hours (0700-1700) then followed up by a repeat survey during the night (1800-0600); both dives were done on the high tide approximately 12 hr apart. Surveys were conducted at one of the no-take sanctuary sites (Fly Point) and one of the non-sanctuary sites (Pipeline) with each site being surveyed on six occasions between October and December 2005. Sites were both sampled within 48 hr of each other. The hypothesis that seahorse abundance would not vary between day and night was tested with a 2-factor analysis of variance (ANOVA) with the factor time treated as fixed with two levels (day, night) and the factor site treated as random and orthogonal with two levels. There was no significant difference in the mean abundance of *H. whitei* between night and day surveys (F_1,24_ = 0.45, *P*>0.5), and the time x site interaction was also non-significant (F_1,24_ = 0.02, *P*>0.5). Therefore, time of surveying was considered irrelevant and all sampling occurred between 0600 and 2200.

### Predator abundance

During 2006, as part of the 60-min monthly surveys and additional dives at the four locations (N = ∼100 dives across four sites), predation events on *H. whitei* were observed and recorded. Species that were classified as predators of *H. whitei* were observed to attack or feed at *H. whitei*. From 2007–2009, during the 60-min monthly abundance surveys (*n* = 36 monthly surveys), the numbers of predators observed at each site were identified and recorded.

### Data analysis

The hypothesis that mean seahorse abundance would differ between the sanctuary zone and non-sanctuary zone sites was tested by 3-factor permutational multivariate analysis of variance (PERMANOVA) using PERMANOVA+1.0.5 within PRIMER-E 6 (Plymouth Routines in Multivariate Ecological Research http://www.primer-e.com/) [33]. The factor Status was analysed as fixed with 2 levels (sanctuary, non-sanctuary), the factor Site was analysed as random with 2 levels and nested in Status, and the factor Year was analysed as random with 4 levels (2006, 2007, 2008, 2009). Each monthly survey was treated as a replicate (*n* = 12) for each year. The analysis was done on the Euclidean distance similarity matrix with significance determined from *n* = 9999 permutations. The same 3-factor PERMANOVA design was applied to test the hypothesis that predator abundance would differ between sanctuary and non-sanctuary sites with the factor Year having 3 levels (2007, 2008, 2009). Post-hoc evaluations of significant results were done using pair-wise *t*-tests. The hypothesis that there would be a relationship between the abundance of seahorses and abundance of predators was tested, using the combined data for all sites from all monthly surveys between 2007 and 2009, by Pearson product-moment correlation coefficient with SPSS 20.

## Results

### Relative abundance of *H. whitei*

**Figure 2:**
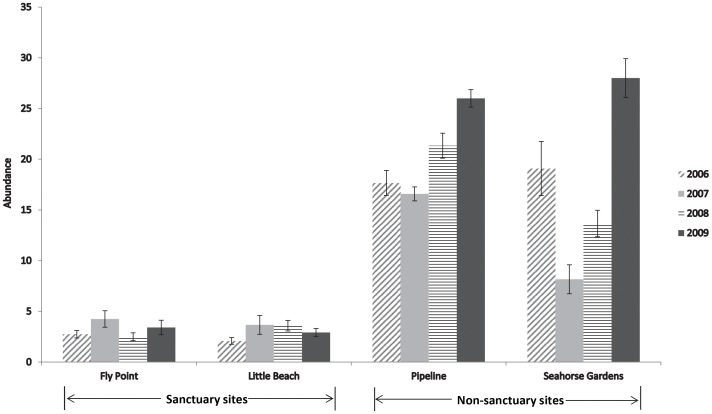
Mean monthly abundance of *H. whitei* (± S.E.) at four sites within Port Stephens for 2006–2009.

A grand total of 2,104 *H. whitei* (1953 adult and 151 juvenile) were observed in the monthly surveys from 2006–2009, with 1802 observed in the non-sanctuary zone (control) sites and 302 observed in the sanctuary zone sites. Mean monthly abundance of *H. whitei* in the sanctuary zone (mean 3.1±0.3 S.E.) was significantly less than the non-sanctuary zone (18.8±0.9) (Table 1, Figure 2) therefore the hypothesis that seahorse abundance differed between the sanctuary and non-sanctuary sites was supported. The significant year x site(MPA) interaction occurred because mean seahorse abundance differed between the two non-sanctuary zone sites in some years but not all years and did not differ between the two sanctuary zone sites in any year (Table 1(a)).

**Table 1:** Summary of hypotheses tested in the long term monitoring of *H. whitei* and predator abundance within and outside a marine protected area (MPA) with details of statistical analysis performed and PERMANOVA results.

Hypotheses	Source	df	MS	Pseudo-F	P(perm)
(a) Seahorse abundance would vary between sanctuary zone sites and non-sanctuary zone sites and years.	MPA site (fixed) Year x Site(MPA)	1	11781	19.67	0.012
	Year (random)	3	409.54	4.19	0.067
	Site(MPA) (random)	2	120.67	1.24	0.358
	MPA x Year	3	483.21	4.95	0.052
	Year x Site(MPA)	6	97.61	6.01	0.001
	Residual	191			
(b) Predator abundance would vary between sanctuary zone sites and non-sanctuary zone sites and years.	MPA site (fixed)	1	2272.11	36.24	0.006
	Year (random)	2	13.03	1.99	0.24
	Site(MPA) (random)	2	61.18	9.33	0.04
	MPA x Year	2	1.69	0.26	0.79
	Year x Site(MPA)	4	6.55	2.17	0.09
	Residual	143			

**Figure 3:**
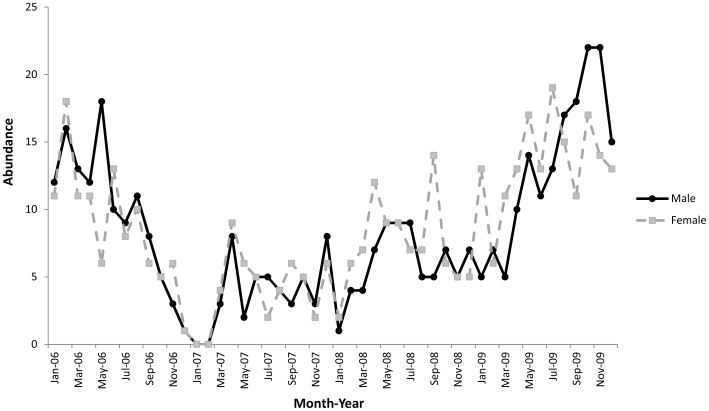
Monthly abundance of adult *H. whitei* recorded in 60 min dive surveys from 2006–2009 at the non-sanctuary site Seahorse Gardens, Port Stephens.

Numbers of *H. whitei* varied greatly at the non-sanctuary sites with a large decline in the *H. whitei* population at the Seahorse Gardens in 2007. The decline commenced in October 2006 and continued until March 2007 (Figure 3), during which the monthly mean abundance of *H. whitei* was 4.8±1.8 compared to the mean monthly abundance of 17.2±1.4 for the site across all years. From January to February 2007, 0 adult *H. whitei* and only 1 small juvenile were observed at the Seahorse Gardens. This was the only time across all four sites and all years when no adult seahorses were observed.

### Predator abundance

**Figure 4:**
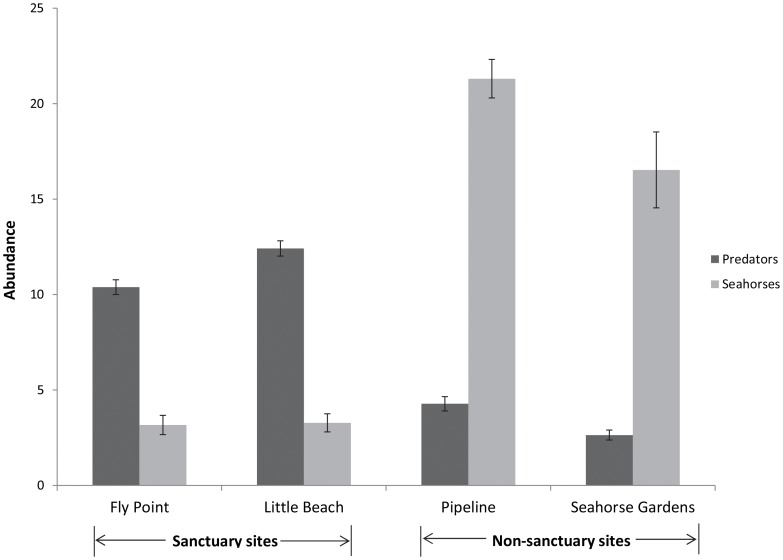
Monthly mean abundance (± S.E.) for *H. whitei* and predators (fish and octopus) for each site from 2007–2009.

**Figure 5:**
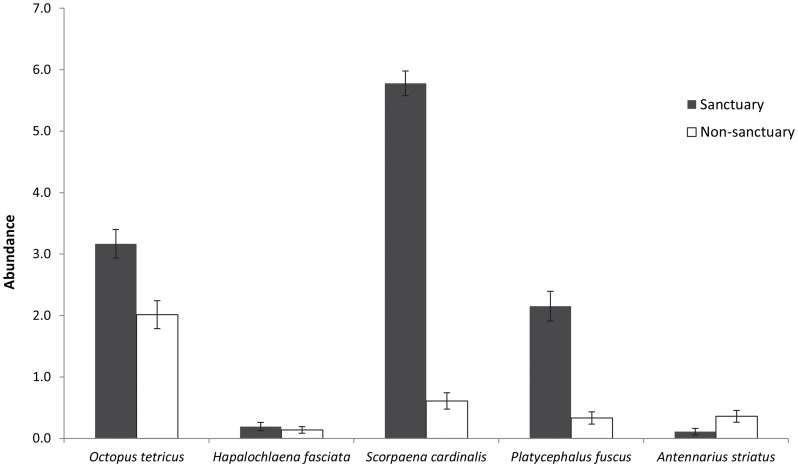
Monthly mean abundance (± S.E.) in 2007–2009 of seahorse predators at two sites within the Fly Point Sanctuary Zone and at two sites outside the Sanctuary Zone.

**Figure 6:**
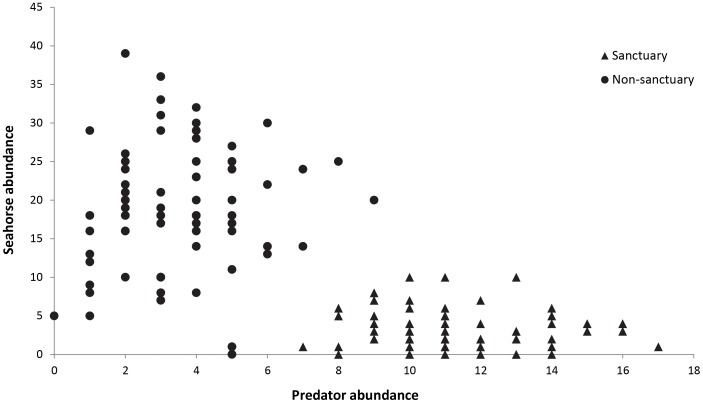
Relationship between monthly seahorse abundance and predator abundance from 2007–2009 at each site.

Five different species preyed on *H. whitei* across the four sites. Three species of fish (dusky flathead *Platycephalus fuscus*, eastern red scorpionfish *Scorpaena jacksoniensis*, and striped anglerfish *Antennarius striatus*) and two species of octopus (Sydney octopus *Octopus tetricus* and blue-lined octopus *Hapalochlaena fasciata*), were recorded either attacking or feeding on *H. whitei.* A total of 13 predation events were recorded from 2006 to 2009 (9 in the sanctuary sites and 4 in the non-sanctuary), with the most frequently observed predation events involving *S. jacksoniensis* (*n* = 5) and *O. tetricus* (*n* = 4). These five species were surveyed monthly from 2007 to 2009 and it was found that the mean number of predators in the sanctuary zone sites (11.4±0.4 S.E.) was significantly greater than the mean number of predators in the non-sanctuary zone sites (3.5±0.3) (Table 1 (b), Figure 4). Therefore, the hypothesis that predator abundance differed between the sanctuary and non-sanctuary sites was supported. The significant Site(MPA) effect occurred because mean predator abundance differed between sites in the non-sanctuary zone but not between the two sites in the sanctuary zone. The most abundant predators in the sanctuary zone sites were *S. jacksoniensis, O. tetricus* and *P. fuscus* (Figure 5). There was a significant, negative correlation between monthly seahorse abundance and predator abundance (*r* = −0.69, *n* = 144, *P*<0.001; Figure 6), with high abundance of predators associated with lower abundance in seahorses.

## Discussion

### Seahorse abundance

The main finding of this study that the abundance of seahorses was significantly lower within a no-take MPA, compared with sites open to fishing, was unexpected as it has been suggested that seahorse abundance would benefit from small-scale no-take MPAs [10]–[12], [17]. The most likely cause of this was the greater risk of predation in the no-take sanctuary zone sites, as suggested by the greater abundance of seahorse predators in these sites and the negative correlation between predator abundance and seahorse abundance. In a study on marine reserves in the Philippines, it was found that syngnathid abundance was lower within the no-take MPA compared to the fished sites outside; however, the difference was not significant and possibly confounded by differences in habitat [34]. In this example, the authors acknowledged that it was difficult to determine if observed effects were real responses to changed increases within the protected MPA (e.g. increased predator abundance) or other factors such as increased species visibility in the non-protected MPA sites as result of less structurally-complex habitats [34].

It is unlikely that the significant difference in *H. whitei* abundance between the no-take sites and control sites was related to habitat differences. Habitat types did not differ between sites and Fly Point, one of the sanctuary zone sites, had the greatest coverage of sponge habitat; a known habitat for *H. whitei* [24]. The observed differences between the no-take and control sites are also unlikely to have been confounded by differences in the detectability of seahorses. Although seahorse detectability may differ among habitat types, there is no evidence that the occurrence of cryptic behaviour among seahorses differed amongst the sites (Harasti *unpublished data*).

One of the often-stated goals of MPAs is the preservation of areas with species and assemblages occurring in an undisturbed state, at least from the exclusion of fishing pressure, for the benefit of scientific research, education and public awareness [35], [36]. Seahorses and other syngnathids are charismatic species that attract support for marine conservation [6], [37]. The findings of this study suggest that seahorses might not benefit from the use of MPAs for marine conservation; however, the finding of this study linking decreased seahorse abundance with increased predator abundance is based on correlative evidence. There are no data available on the abundances of seahorse and their predators prior to the establishment of the no-take MPA. To test and validate this finding, field experiments are needed to determine the actual rates of seahorse predation between sites closed and open to fishing, and to determine other predator species [38],[39].

### Predators of Hippocampus whitei

As seahorses are a slow-moving species, they rely on crypsis through colour changes and algal-like filaments that mimic their habitat, to avoid predation [24], [40]. Eighty-two predators of syngnathids are known, including fishes, turtles, sea birds, invertebrates and marine mammals [41]. None of the predators recorded in this study were included in the [41] review, with the only recorded predator of *H. whitei* in the literature being the little penguin *Eudyptula minor* whilst [24] observed the striated frogfish *Antennarius striatus* predating on *H. abdominalis*, a seahorse known to occur in the same region as *H. whitei* [29].

The cephalopod *Octopus tetricus* and the scorpionfish *Scorpaena jacksoniensis* are believed to the most frequent predators of *H. whitei* as they were responsible for the majority of observed predation events and were the two most abundant predators. However, given the large diversity and size of fishes found within the Fly Point no-take MPA site [42], there are potentially other predators of *H. whitei* that were not detected. During monthly surveys from 2008–2009, both snapper *Pagrus auratus* and leatherjacket *Nelusetta ayraudi* were observed to attack *H. whitei* following their release after being handled underwater (for measuring or tagging as part of other studies). This occurred if the seahorse swam away from the holdfast it was placed on after handling. However, there were no observations of either species attacking *H. whitei* that had not been ‘disturbed’. Another cephalopod species, the mourning cuttlefish *Sepia plangon*, was observed to prey on juvenile *H. whitei* on two separate occasions within the sanctuary zone; however, it was not included in the monthly predator surveys as the observations occurred in 2008 and 2009, prior to the predator study reported here.

### Predator abundance

Seahorse predators were more abundant within the no-take sanctuary zone sites, which is similar to findings of other studies from around the world that have reported greater abundance and/or biomass of predator fishes in areas protected from fishing [34], [43]–[45]. The three most abundant predator species (*Platycephalus fuscus*, *Octopus tetricus* and *Scorpaena jacksoniensis*) were more abundant within the sanctuary zone and are considered to be important recreational and commercial species that are targeted by fishers in NSW [46], therefore these species are likely to benefit from the exclusion of fishing. Additionally, data collected from baited underwater remote video systems has found that the Fly Point sanctuary zone has greater diversity and larger fish species than the non-sanctuary zone sites (NSW DPI *unpublished data*). This is also supported by the findings of Edgar *et al*. (2009) that demonstrated that Fly Point was high in fish biomass and in density of larger fish species. The increased numbers of predators within the sanctuary zone sites is not surprising, as the sanctuary zone has been protected for 30 years (since 1983) with no fishing allowed, and numerous studies have shown that fish biomass and density increased over time within MPA's [13], [15], [42]–[45]. With the implementation of MPAs, there will be ‘winners and losers’, with some species benefiting from protection by increases in size and abundance [47], [48]. Other species showed no change in abundance or abundance decreased as a result of increased predation and interspecific competition [28], [49], [50], particularly the smaller cryptic fishes [34], [51], [52]. Protected areas have been shown to help promote recovery of predatory species [43], [53], which potentially can have indirect negative effects on prey species in the protected areas [28].

Whilst this study suggests that *H. whitei* has been negatively impacted by a no-take protected area most likely through increased predation, other species of seahorse and other syngnathids might be affected in different ways by MPAs. Species' responses to MPAs will depend on a range of factors including the availability of preferred habitats, potential predators in the area and factors occurring outside an MPA.

### Decline in *Hippocampus whitei* abundance

Population estimates and monthly relative abundance data show that *Hippocampus whitei* populations across the four sites in Port Stephens were stable with the exception of the Seahorse Gardens, which experienced a large population decline in late 2006. As the species is protected and not exploited by fishing, such an abrupt decline is unusual and the cause of the decline is unknown. Population declines in *Hippocampus sp.* in the absence of fishing pressure have been recorded elsewhere, with *H. abdominalis* populations declining 79–98% over 3 years [54] and populations of *H. guttulatus* and *H. hippocampus* declining by 94% and 73%, respectively over a seven year period [55]. Given that the decline of *H. whitei* in this study occurred only at the Seahorse Gardens site, it is unlikely that the decline can be attributed to ecosystem-wide stressors such as disease or environmental variables as populations at the nearby three sites should also have been affected. Throughout the study, there was no noticeable change in currents or water temperature at the Seahorse Gardens. These two variables were, however considered to influence changes in seahorse abundance in Ria Formosa lagoon [55]. There were also no recorded incidents of illegal collecting, nor was the site affected by trawling, netting or dredging which are prohibited in the area. Seahorse predator abundance at the site did not increase during the study, nor were there observations of increases in other species that may prey on *H. whitei.*

A potential hypothesis for the decline is that the seahorses may have moved off the site into deeper water; however, *H. whitei* is known to have small home ranges and displays site fidelity [17], [22]. Support for the movement hypothesis is that several seahorses tagged at the site in 2006, disappeared during the decline period, but started to be resighted again from late 2007 until 2010 [23]. Numerous exploratory dives were undertaken in the deeper water (12–18 m) surrounding the Seahorse Gardens site from 2006–2009; however, no *H. whitei* were encountered deeper than 12 m (maximum depth of study site), so the location to which seahorses might have migrated is unknown. With such a population decline there is concern that reproduction would be reduced as a result of Allee effects [56], especially with the high level of monogamy displayed by *H. whitei* [23], as mature animals could find it difficult to find a mate. Although there was a rapid decrease in population abundance, the actual recovery of the population to almost pre-decline levels occurred within three years with the highest number of juveniles at the site occurring in 2009. As *H. whitei* is considered an R-selected species with rapid growth, early age at maturity and sexually mature at approximately six months [23], the species has the potential to repopulate a site if sufficient breeding adults return to the site, or recruitment from adjacent sites is successful.

## Conclusions

This study illustrates the importance of long-term monitoring of seahorse populations as it was shown that seahorse numbers varied considerably over a 12-month period. Long-term monitoring of multiple sites is necessary for a good understanding of seahorse population changes in the wild and allows for better assessment on the status of seahorse populations. The study indicates that caution should be used when investigating the use of MPAs to conserve seahorse populations as there is potential for negative impacts on seahorse abundance through potentially increased predator abundance. Other management interventions may be more suitable such as entire protection of the seahorse species, removal of destructive fishing practises that damage essential habitats, restoration of natural habitats or creation of artificial habitats. A range of management measures are needed to conserve threatened populations of seahorses and the declaration of a marine protected area may not be the ideal solution.
